# Shaping Synaptic Learning by the Duration of Postsynaptic Action Potential in a New STDP Model

**DOI:** 10.1371/journal.pone.0088592

**Published:** 2014-02-14

**Authors:** Youwei Zheng, Lars Schwabe

**Affiliations:** Faculty of Computer Science and Electrical Engineering, University of Rostock, Rostock, Germany; The University of Plymouth, United Kingdom

## Abstract

Single spikes and their timing matter in changing synaptic efficacy, which is known as spike-timing-dependent plasticity (STDP). Most previous studies treated spikes as all-or-none events, and considered their duration and magnitude as negligible. Here we explore the effects of action potential (AP) duration on synaptic plasticity in a simplified model neuron using computer simulations. We propose a novel STDP model that depresses synapses using an AP duration dependent LTD window and induces potentiation of synaptic strength when presynaptic spikes arrive before and *during* a postsynaptic AP (dSTDP). We demonstrate that AP duration is another key factor for insensitizing the postsynaptic neural firing and for controlling the shape of synaptic weight distribution. Extended AP durations produce a wide unimodal weight distribution that resembles the ones reported experimentally and make the postsynaptic neuron tranquil when disturbed by poisson noise spike trains, while equivalently sensitive to the synchronized. Our results suggest that the impact of AP duration, modeled here as an AP-dependent STDP window, on synaptic plasticity can be dramatic and should motivate future STDP studies.

## Introduction

Synaptic plasticity is sensitive to the timing of pre- and postsynaptic firings. Ever since the first experiments demonstrating that Hebbian synapse exists [1] and first recordings revealing that the coincidence of postsynaptic action potentials (APs) and excitatory postsynaptic potentials (EPSPs) was sufficient to induce long-term changes in synaptic efficacy [2], many experimentalists and theoreticians believed that “timing is everything”. These findings led to a generally accepted phenomenon known as spike timing-dependent plasticity (STDP) [3], [4] even though its generality has been questioned because STDP-induced synaptic modification is contingent upon many other factors [5], [6]. Despite ongoing debates over STDP as a general model for synaptic plasticity, the key principle of STDP is still thought to be the pairing causality [7].

APs, or the backpropagating signals triggered by APs, are believed to play the most crucial role in STDP. However, theoretical studies usually treated spikes as all-or-none events, with the duration and magnitude of which not being taken into consideration. Thus, it is no surprise that the functional role of AP duration or magnitude on STDP has never been investigated, neither experimentally nor theoretically. It is worth noting that AP duration differs between cell types. GABAergic interneurons often [8], but not always [9] or exclusively [10], have shorter AP durations than pyramidal neurons. In addition, AP duration is widely used to identify dopamine (DA) neurons and it was shown that the projection targets of DA neurons correlate with their AP durations, for instance, nucleus accumbens-projecting neurons may have a duration of 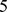 ms, which is almost twice as long as for amygdala-projecting ones [11]. Even within the same neuron type, what’s more worth noting is that AP duration, which is generally accepted as a stereotypic property, can be modulated via BK channels [12]. All above evidences led us to the hypothesis that such differences are not accidental but may play a role in information processing, learning, memory and even in certain disease models. Besides, since AP broadening may exert a significant impact on various types of calcium channels that can lead to an increase of calcium entry and thus favor a strengthening of synaptic conductance [13], AP duration may have an impact on synaptic plasticity that is not negligible. Elucidating this impact will definitely shed light on the mechanisms underlying how synaptic plasticity shapes cortical networks of excitatory and inhibitory neurons, and how various projection pathways of DA and non-DA neurons differ, e.g. given an identical plasticity protocol.

Our simulation study was set up to investigate the impact of AP duration on synaptic plasticity, in which AP duration is incorporated into a canonical pairwise STDP model. First, we report that a recently proposed voltage-dependent STDP model [14], [15] depends on AP duration and magnitude. Motivated by these findings we formulate in the spirit of simplicity a novel STDP model that includes an AP duration- dependent window function. The new model is operated then in both additive and mixed modes, and its predictions are compared with previous canonical models.

## Results

### AP Dependence of a Voltage-dependent STDP Model

A recently proposed phenomenological model accounts for a variety of experimental findings [14]. This model is voltage dependent and does *not* treat spikes as all-or-none phenomena, from which the predicted synaptic modifications depend not only on the plasticity model but also on the neuron model. We first replicated the simulation results from [14] and then varied AP duration. Two typical STDP experimental protocols were simulated, an interspike interval (ISI) protocol and an interspike frequency (ISF) protocol, respectively with a set of AP durations.

**Figure 1:**
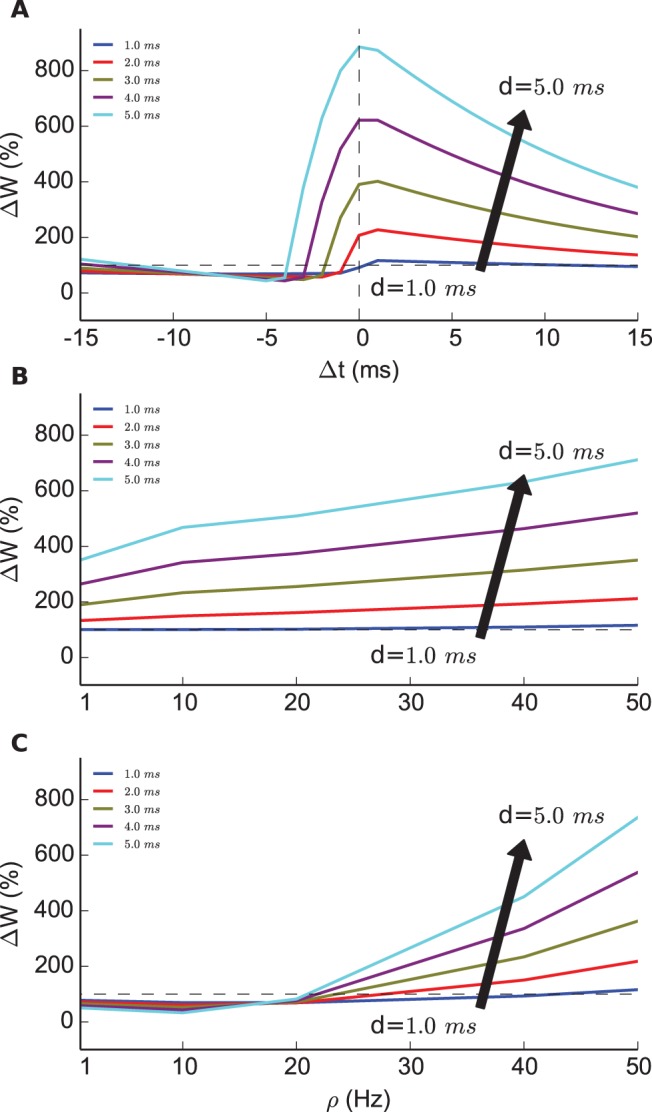
Synaptic modification by a voltage-dependent STDP model. **A.** STDP windows generated by stimulating the model with 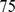 pairs (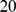 pairs per second) of pre- and postsynaptic spikes with 5 different pairing intervals 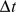 (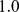 to 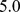 ms). (**B,C**) The relative peak modification of synaptic weight simulated by different pairing frequency 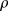 with positive pairing interval 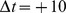 ms (pre-post) and 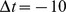 ms (post-pre).

Figure 1A shows the STDP curves obtained from the ISI protocol with 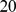 pairs per second, and different interspike intervals 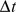. We observe that the longer the AP duration the more the predicted STDP window is up stretched. In particular, a synapse is potentiated for long APs even when presynaptic spikes arrive within, which resembles the curves reported in [16]. The peak potentiation is higher for long AP durations, because at pre-post pairings they keep the membrane potential at higher voltage, which in the model translates into more potentiation.

We find that AP duration affects as well the predicted synaptic modifications under the ISF protocol. Potentiation is predicted to increase with higher frequencies of pre-post pairs, 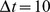 ms, for longer but not for short APs (Figure 1B & C). Altogether we confirmed that AP duration of 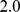 ms produced the model predictions, that are in agreement with previous experiments [17].

These results emphasize the importance of the duration of postsynaptic AP in synaptic plasticity. Two predictions which we include below into our new model are: i) presynaptic spikes strengthen a synapse when they arrive before and during an AP, but they weaken a synapse when arriving afterwards, and ii) the magnitudes of these modifications depend on AP duration. In support of these model predictions, one very original STDP paper did show some data points, illustrating positive synaptic weight changes given negative 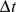 [18].

### Motivation and Formulation of dSTDP Model

When modeling synaptic plasticity one could focus on at least two important aspects, namely the detailed biophysical/biochemical dynamics and the emergent functional properties of learning rules. We decided to focus on the latter and defined a simple phenomenological model to study how AP duration affects the learning in model neurons. In this model individual synapses do *not* interact directly with each other. However, via the spiking of the postsynaptic neuron the synaptic strengths of individual synapses become dependent on each other. For example, an early modeling study [19] demonstrated how competition could arise in this way.

Our approach is to first understand how AP duration may affect the pattern of synaptic strengths before investigating the biophysical/biochemical properties, because the modeling studies are currently not conclusive. Experimentally characterized STDP window exhibits a sharp transition at 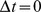 ms, where differences of only a few milliseconds determine whether LTP or LTD is induced. Previous models that could account for the sharp transition at 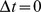, are not fully compatible with the available data from STDP experiments. For example, an afterdepolarization (ADP) model [20], which suggested that STDP was strongly dependent on the magnitude and duration of the ADP, could explain the transition at 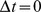 ms. It predicts LTP for 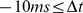 for an ADP of 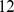 mV (but not for no ADP), which is not unlike our simulation with the model from [14]. However, it predicted LTD for longer positive 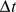, which to the best of our knowledge has never been reported before. Another model that considered the duration of backpropagating action potential [21], predicted a scaling of STDP function similar to our simulations (Figure 1A). This model also predicted LTD for longer positive 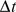, and in addition failed to reproduce plausible STDP curves when pairing frequencies become larger. We conclude that at the current stage of STDP modeling, an exploration on how AP duration and magnitude affect the emergent properties of synaptic plasticity may be best conducted with phenomenological models.

**Figure 2:**
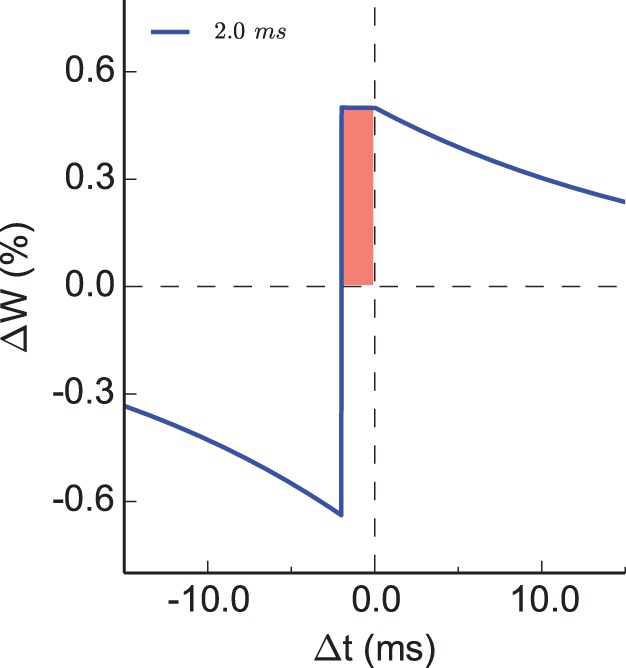
dSTDP window function. The relative modification of synaptic weight 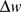 varies as a function of interspike interval 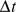 (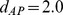 ms).

We study two key functional consequences of STDP, the shape of synaptic weight distribution and the regulation of postsynaptic spiking rate [3]. The model is based on three assumptions: first, a synapse is potentiated when a presynaptic spike arrives before *and* during a postsynaptic AP; second, a synapse with a presynaptic spike arriving after the postsynaptic AP is depressed with a magnitude depending on AP duration in order to control the overall LTP/LTD ratio; third, the effect of AP duration is uniformly distributed through the length, modeled via a plateau in the STDP window. We construct the model in such a way that it can be simulated in both additive [19] and mixed modes (additive update for potentiation and multiplicative update for depression, see details in [22]). For AP duration is explicitly included within the model, we name it dSTDP. More specifically, given a presynaptic spike at 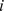-th excitatory synapse and a postsynaptic spike elicited by an interval 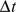, the corresponding change of the synaptic weight 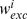 is illustrated in Figure 2 and defined as:
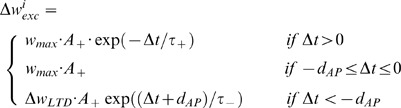
(1)where 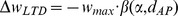 is used in the additive mode and 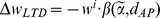 in the mixed mode. The AP duration dependent term 
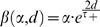
 is introduced to keep the ratio of positive integral to negative integral equal to constant 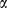 (Figure S1). This procedure is intended to eliminate significant changes in the ratio of LTP/LTD areas induced by AP duration, which could be a potential confounding factor [19]. All important parameters are listed in Table 1. and an all-to-all pairing scheme is implemented to update synaptic modifications throughout [23].

**Table 1:** Neuronal, synaptic and plasticity parameters.

Parameter		Symbol		Default value
Membrane capacity		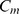		200 pF
Leak conductance		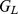		10 nS
Membrane time constant		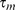		20 ms
Spiking threshold		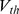		–54 mV
Resting membrane potential		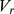		–70 mV
Reset membrane potential		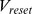		–60 mV
Adaptive reversal potential		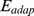		–70 mV
Adaptation time constant		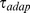		100 ms
Action potential duration		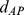		2.0 ms
Synaptic time constant		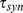		5 ms
Potentiation time constant		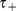		20 ms
Depression time constant		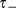		20 ms
Inhibitory synaptic strength		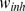		500 pS
Number of excitatory synapses		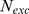		1000
Number of inhibitory synapses		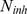		200
Excitatory input rate		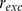		10 Hz
Inhibitory input rate		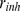		10 Hz
Maximum potentiation amplitude		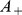		0.005
Learning ratio in additive mode		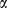		1.05
Learning ratio in mixed mode		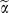		2

### Comparison of Equilibrium Weight Distributions

**Figure 3:**
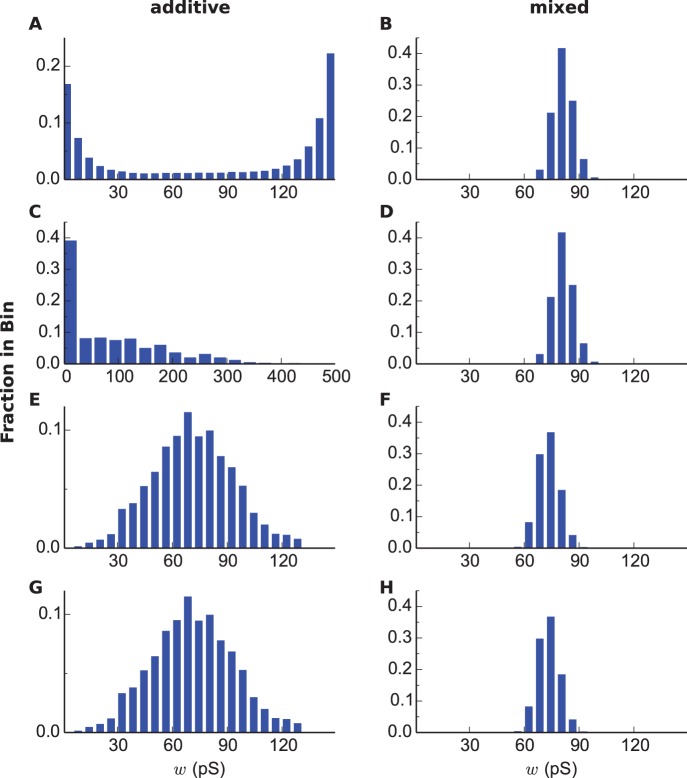
Equilibrium synaptic weight distributions for dSTDP models. (For A,B,C,D, 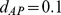 ms.) **A:** A U-shaped bimodal distribution generated by additive mode, with an upper-bound. **B:** A centered unimodal distribution generated by mixed mode, with an upper-bound. **C:** Similar to A, without an upper-bound. In this scenario, the distribution didn’t equilibrate as in A, the data was taken at 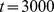 sec. **D:** Similar to B, without an upper-bound. (For E,F,G,H, 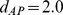 ms.) **E:** A wide unimodal distribution generated by additive mode, with an upper-bound. **F:** The slightly skewed centered unimodal distribution generated by mixed mode, with an upper-bound. **G:** Similar to E, without an upper-bound. **H:** Similar to F, without an upper-bound.

The dSTDP model was simulated together with an integrate-and-fire model neuron whose activity was driven by both excitatory and inhibitory poisson spike trains (see Methods). We first re-examined the equilibrium synaptic weight distribution for short AP duration 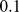 ms (equivalent to the model with a canonical STDP window, e.g. [19]) in both modes. With synaptic weights tamed by an upper-bound, the additive mode produces a U-shaped bimodal distribution (Figure 3A), well matching the results reported in [19]. The weight-dependent mixed mode generates a centered unimodal distribution (Figure 3B) in which the synaptic weights are very narrowly distributed, consistent with a previous simulation study [24].

As previously contemplated, when simulated without imposing an upper-bound, the additive mode has an inherent instability in that a few synapses get boundlessly stronger due to a destabilizing force, while the others become weaker (Figure 3C). By contrast, the mixed mode has an intrinsic stability and produces the very same distribution, independent of the existence of an upper-bound (Figure 3D), because the effect of the destabilizing force is relatively small as the stabilizing force dominates, which then constrains the weight growth [25].

For simulations of dSTDP models we chose an AP duration of 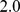 ms long. Interestingly, dSTDP in the additive mode predicts a wide unimodal distribution (Figure 3E), which clearly differs from the U-shaped produced by the canonical model (Figure 3A). This difference can be understood as follows: the additional potentiation force introduced by the AP duration counters the extra depression induced by the 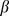-term. As a consequence, independent of the initial synaptic weights, most of the synapses tend to stay in the middle range of the weight spectrum (Figure S2). Note that the distribution remains stable without an upper-bound (Figure 3G), indicating an intrinsic stability property possessed by the model. The dSTDP model simulated in the mixed mode predicts a similar narrow unimodal distribution, but slightly skewed(Figure 3F & H).

### AP Duration Determines the Shape of Equilibrium Synaptic Weight Distribution

**Figure 4:**
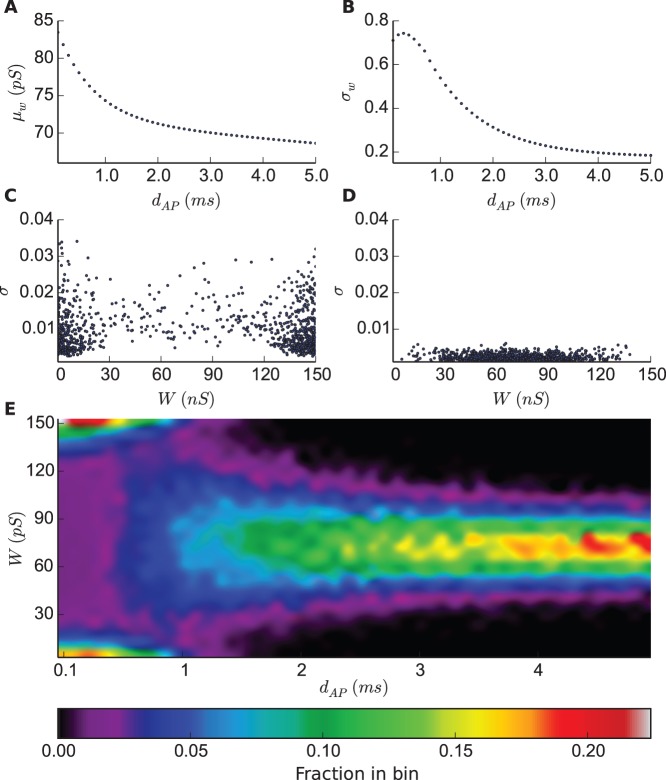
AP duration determines the shape of equilibrium synaptic weight distribution (additive mode). **A:** The average of excitatory synaptic weights given various AP durations. **B:** The standard deviation of excitatory synaptic weights given various AP durations. **C:** The standard deviation of each individual synaptic weight for an 1000-sec post-equilibrium simulation run (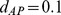 ms). **D:** Same as in C, 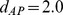 ms. **E:** The horizontal axis is AP duration, the vertical axis is synaptic weight and color bar indicates the probability density.

The results shown in Figure 3 suggested to us that equilibrium synaptic weight distribution could be largely attributable to the length of AP duration in dSTDP models. Therefore, we simulated dSTDP model in the additive mode with an upper-bound 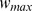 for various AP durations, ranging from 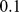 to 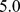 ms, and observed the resultant distributions illustrated in Figure 4E. The weight distribution loses its bimodal shape as AP duration increases and transitions to a complete unimodal for durations that are larger than approximately 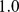 ms, and it becomes even narrower for larger ones. Accompanied with the reshaping of the distribution, the average and the standard deviation of synaptic weights decrease for longer AP durations (Figure 4A & B), which were computed from the histograms shown in Figure 4E. They illustrate one experimental prediction of dSTDP model: the postsynaptic AP duration is predicted to be inversely correlated with the average synaptic strength as well as its variability, which could be experimentally measured using, for example, ensemble statistics of spontaneous miniature or evoked EPSPs.

Note that even though the distribution reaches an equilibrium state, the individual weights keep fluctuating as the simulation goes along. The fluctuation for short AP duration (Figure 4C, 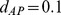 ms), is around one order of magnitude larger than for longer ones (Figure 4D, 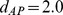 ms). This reflects the difference between the bimodal and unimodal weight distributions as for instance in the former, strong synapses can become weak and vice versa, which results in large temporal fluctuations. In comparison, the mixed mode exhibits fairly small variations in both statistical properties and hence doesn’t result in significant AP duration dependent changes in equilibrium synaptic weight distribution (Figure S3).

### Postsynaptic Response to Signal and Noise Inputs

In this modeling study, most of the presynaptic inputs were modeled as poisson spike trains, similar as what was conducted also in some previous works [19], [26], [27]. While it is still not clear if in the real brain such poisson spikes carry relevant information or should rather be considered as a source of background noise, recent evidences suggested that correlated synchronous neural activity is informative about the features of stimulus in the early sensory processing [28] as well as about behavioral states [29]. Therefore, we were interested in the role of AP duration in dSTDP for processing inputs that are composed of both synchronous spikes as the signal and poisson spikes as the noise.

**Figure 5:**
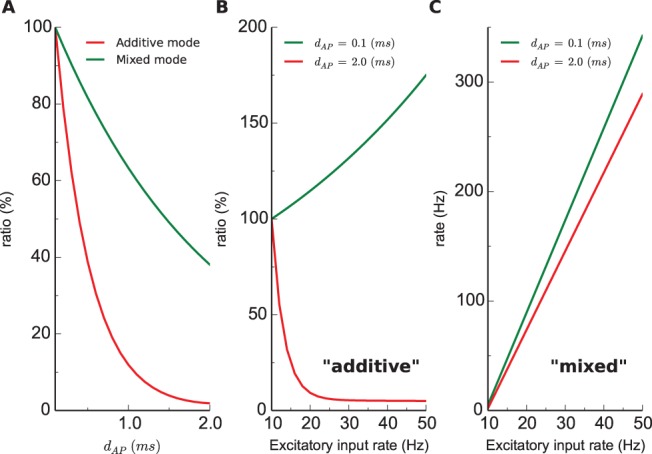
Regulation of postsynaptic spiking rate by AP duration (noise). **A.** The ratio of steady-state rates for various AP durations, referenced to the maximum with 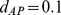 ms. **B.** The ratio of steady-state rates for various excitatory input rates, referenced to the rate when both excitatory and inhibitory inputs are 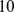 Hz (additive mode). **C.** Similar as B, the *actual* steady-state rates are plotted (mixed mode).

As done above for investigating the synaptic weight distributions, we first explored the effect of AP duration on regulating the postsynaptic response to the noise. The fluctuation analysis above (Figure 4C & D) suggests that the dynamics of the postsynaptic neuron must also undergo a big change. It has been discovered that given poisson noise, STDP rules have remarkable effects on regulating the long-term average spiking rate of the postsynaptic neuron [19], [30] as the synaptic weight distribution converges to an equilibrated state. We find that dSTDP models also possess such a regulation of the postsynaptic spiking: 1) the rate simulated with the additive mode dSTDP decreases quickly as AP duration extends (normalized ratio in Figure 5A, red); 2) such effect is as prominent with the mixed mode, the rate drops almost by 60% when varying AP duration from 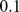 ms to 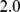 ms (Figure 5A, green), despite the fact that the weight distribution has only a small shift to the *left* (Figure 4B & F). Analyzing a selected simulation run shows that the absolute asymptotic postsynaptic spiking rate for a long AP duration is much lower compared to a short one (0.1 vs 7.5 Hz, see representative traces in Figure S4). Systematic simulations reveal that AP duration exerts a considerable effect on the steady-state spiking rate, making the model neuron much insensitive to the noise (Figure 5A & B). Such regulation is expected as the weight distribution narrows (Figure 4E) and total weight of synapses decreases when AP duration is elevated (Figure 4A).

A recent model demonstrated an interesting regulation of the steady-state postsynaptic spiking, namely that the rate has a non-monotonic dependence on the level of excitatory noise [26]. It exhibits by dSTDP models a distinct monotonic functional dependence, affected by the length of AP duration. We find that in the additive mode: 1) when AP duration is short, the postsynaptic spiking rate rises from 7.5 Hz to about 13 Hz as the excitatory input rate is elevated from 10 Hz to 50 Hz (Figure 5B, green). The result is consistent with the previous study, as each 5 Hz increase causes an elevation of the output rate roughly by 1 Hz [19]; 2) when AP duration is long, however, an exponential-decreasing dependence was observed (Figure 5B, red), which can be understood intuitively as the postsynaptic neuron shifts most of the synapses to weaker strengths for higher presynaptic activities (Figure S5). Such “buffering” effect is much weaker in the mixed mode (Figure 5C) and the rate undergoes a more than 60-fold increase from 5 Hz to 300 Hz for just a 5-fold increase of the input rate, but still, the increase is less pronounced for longer AP durations (Figure 5C, red).

**Figure 6:**
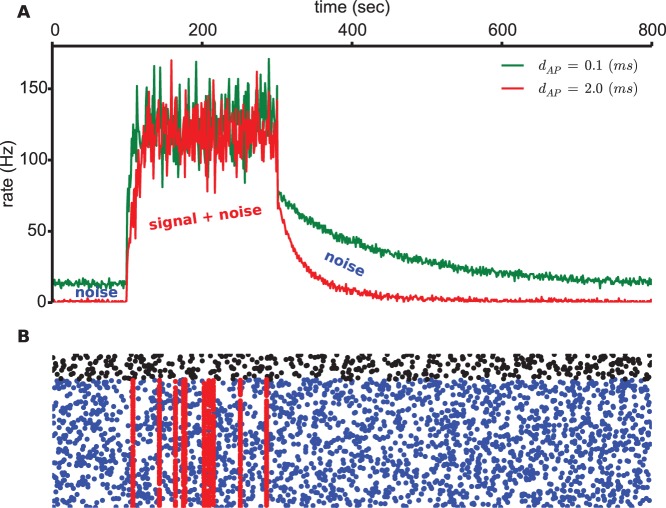
Sensitivity of postsynaptic spiking rate to synchronized spikes. **A.** Driven by the presynaptic spike activity illustrated in B, the rates are shown for two different AP durations. **B.** A raster plot of poisson spike trains (noise) and synchronized spikes (signal). The blue dots are the excitatory inputs (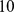 Hz), the black ones the inhibitory (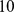 Hz). Synchronous spikes are represented by the red dots which occur on the excitatory synapses only (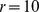 Hz and 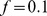).

Then we simulated a scenario where the model neuron was first driven by the noise alone. The weight distribution converges towards an equilibrium before the signal was applied for 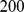 seconds. After removing the signal the weight distribution re-equilibrated again (Figure S6). Interestingly, we observe that postsynaptic spiking remains equally sensitive to the signal (plateau phase), while the noise is much more effectively filtered (decay phase) by a long AP duration than a short one (Figure 6).

### Simulations of Unbounded dSTDP

**Figure 7:**
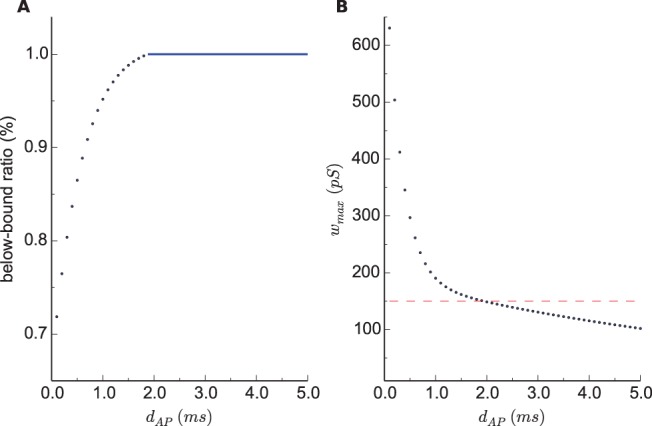
dSTDP models unbounded. **A:** The ratio of synaptic weights below 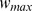. **B:** The maximum synaptic weight obtained throughout each simulation run (3000 sec).

We have shown that in the additive mode without an upper-bound, short AP duration leads to a development in which a number of very strong synapses continue to grow their strengths way beyond 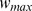 (Figure 4C). Taking a snapshot at time 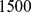 sec in the simulations, we observe that on average only 72% of the excitatory synapses have weights smaller than or equal to 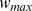. We find that this below-bound ratio increases as AP duration is extended (Figure 7A), for instance, all synaptic weights are constrained below 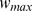 once AP duration is larger than 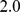 ms. Moreover, we also picked out the maximum synaptic weights for various AP durations from simulations (each ran for 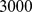 sec). The maximum decreases when AP duration is prolonged and no single weight exceeds 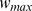 when APs are longer than 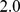 ms (Figure 7B). These results confirm from a distinct perspective that AP duration has an inherently stabilizing effects which should motivate further studies.

## Discussion

The main contribution of this paper is to emphasize that AP duration is an important and so far poorly investigated feature of STDP. Here we refer to the emergent properties of AP duration in a model neuron driven by a number of excitatory and inhibitory poisson noisy spike trains as well as the synchronized on the synapses, *not* its effects in microscopic models of synaptic plasticity [20], [21] or signal transduction pathway models of a postsynaptic spine [31]. In our simulation study we used a recently proposed unified model [14] as the basis for our novel simplified linear dSTDP model, which includes AP duration directly into a STDP window function.

This dSTDP model in the additive mode makes several unique and testable predictions: i) the synaptic weight distribution depends on AP duration with a bimodal shape for short ones and a unimodal for the longer (Figure 4E); ii) the mean and standard deviation of this distribution decrease for elevated APs (Figure 4A & B). It is worth noting that this prediction is in good consistency with the data shown in some previous experimental works, for instance the duration of back-propagating action potential typically increases on the dendrites with the distance from soma [32] which may account for a decremental average response observed in the distal compartments [33]. Besides, our results are also in agreement with another simulation work that modeled the effect of AP duration as axonal delay [34], this work showed also a decrease in summed synaptic strengths after STDP training; iii) the fluctuations of individual synaptic weights over time depend on AP duration with stronger fluctuations for the short (Figure 4C & D); iv) the model neuron with long APs is able to filter out most of the poisson noise input while remains very sensitive to the modeled signal (Figure 6).

Given that our starting point was a phenomenological model itself [14], one could question the validity of our simulation study. However, we used this model as our starting point, because it is a model from which the plasticity outcome is *directly* determined by the duration and magnitude of the postsynaptic AP, whereas the prediction from more complex models may be indirect, for instance, depending on the modeling of calcium concentration [21] or kinetics of NMDA receptors [35]. Moreover, the dSTDP window we postulate here is consistent with the published experimental data, which is itself rather noisy and does not fully constrain the window function at the transition between potentiation and depression.

One could argue that neither a narrow unimodal [24] nor a bimodal weight distribution are of functional interests [19], and a stable *Gaussian* distribution should be the goal of modeling studies [25]–[27]. This contradicts recent experimental observation that reported a unimodal weight distribution with long tail (“a few strong connections immersed in a sea of weaker ones” [36]). Interestingly, we did observe such a distribution in a version of dSTDP model which does not have the control for the ratio of integrated LTP/LTD windows (data not shown).

Studying how AP duration affects the emergent properties of synaptic plasticity in a single neuron or neural networks, is certainly a field of interest for both theoreticians and experimentalists. Sixty years after Hodgkin and Huxley’s original publication, the action potential (its shape and duration) should get renewed attention in particular from the field of synaptic plasticity and this simulation work may qualify as yet another candidate model in the spectrum of STDP modeling to be further explored, analytically and experimentally.

## Methods

### Neuron Model

The model neuron receives 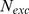 excitatory and 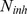 inhibitory poisson spike trains [19], [25]–[27], [37], [38]. A standard single-compartment conductance-based leaky integrate-and-fire neuron with a spike-triggered adaptation current [38] is used as to simulate the dynamics of membrane potential 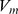:
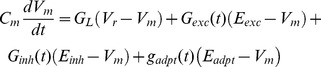
(2)where 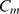 is the membrane capacity and 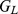 is the leak conductance. When the membrane potential reaches the threshold 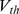, the model neuron spikes an AP. To cooperate with the dSTDP model we introduce 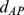 as the new parameter, denoting AP duration, such that when the membrane potential reaches the threshold from below at time 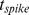, we assign 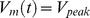 for 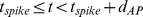 and then reset 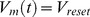 at 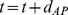. This is certainly a gross simplification for modeling the effects of AP duration, but we introduce this simplistic parameterization in order to obtain a first qualitative characterization of its impact on synaptic conductances via synaptic learning.

The adaptation conductance 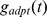 increments by 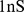 after each postsynaptic spike, namely at the time of threshold crossing 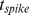, and then decays with a time constant 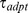. It models spike-frequency adaptation due to, for example, calcium-dependent potassium currents. The total synaptic conductances 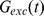 and 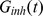 represent summed contributions from all excitatory and inhibitory synapses. Whenever a presynaptic spike arrives at the 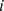-th synapse, the corresponding total conductance is increased instantaneously by 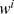 and then decays with a time constant 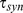. All inhibitory synapses have an unmodifiable strength 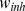, whereas 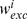 is updated by STDP learning.

### Construction of Synchronous Spikes

For the spike trains used in Figure 7 and Figure S6, synchronous events are generated in a poisson manner. More specifically, we define a rate 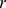 and a fraction 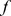 of the presynaptic spikes being synchronized, then within each simulation time bin 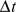, the probability of a synchronous event to occur is 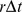, and on every occurrence of such synchrony, 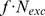 presynaptic excitatory spikes are synchronized.

## Supporting Information

Figure S1
**The AP duration dependent term 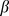.** The peak synaptic modification for depression is modeled as a function of AP duration, 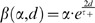.(EPS)

Figure S2 **Differential developments of synaptic weights.** By short AP duration, additive mode drives most of the synapses either to a potentiated state (green) or a depressed state (blue), resulting in a bimodal distribution. However, by long AP duration, the most stay in the middle (red).
(EPS)

Figure S3 **AP duration has little effect on shaping synaptic weight distribution in the mixed mode.** Similar as illustrated in Figure 4, but simulated with mixed mode.
(EPS)

Figure S4
**Postsynaptic spiking rate traces.** The rates are calculated using a bin of one second for 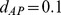 ms and 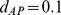 ms.(EPS)

Figure S5
**The effect of elevated excitatory noise input on equilibrium synaptic weight distribution and postsynaptic spiking rate (additive mode). A & B:** The distribution and rate taken from 1000 sec after equilibrium, 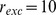 Hz. **C & D:** Similar as in A & B, 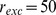 Hz. (The inhibitory noise input rate was kept unmodified).(EPS)

Figure S6
**The effect of synchronous signal input on synaptic weight distribution (additive mode).** **A1:** All the excitatory synapses have an initial weight of 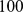 pS. **A2:** The equilibrium synaptic weight distribution converges to a bimodal shape after receiving noise only. **A3:** When afterwards receiving both noise and signal, most of the synapses are strengthened towards 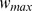. **A4:** The removal of the signal re-equilibrates the distribution back to a bimodal shape. **B:** A raster plot represents the presynaptic spike trains, aligned with the different stages that cause the change of synaptic weight distribution. **C:** All panels are similar to A, but simulated with AP duration 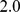 ms.(EPS)
